# Supplementary material for: Agrochemicals increase risk of human schistosomiasis by supporting higher densities of intermediate hosts
Source: Nat Commun. 2018 Feb 26;9:837. doi: 10.1038/s41467-018-03189-w (PMC5826950; doi:10.1038/s41467-018-03189-w)
Supplement: Supplementary file 1 — Supplementary Information [file 41467_2018_3189_MOESM1_ESM.pdf]

Agrochemical pollution increases risk of human exposure to schistosome parasites

Halstead *et al.*

Supplementary Information

Supplementary Methods

Supplementary Figures 1-5

Supplementary Tables 1-10

Supplementary References

Supplementary Methods

Effects of Alternative Food Sources for Predators and of Snail Refugia on Snail Densities

To explore the potential for alternative food resources for omnivorous predators and the presence of refugia on snail densities, we used data from a follow-up mesocosm experiment on the individual effects of different classes of herbicides and insecticides to opportunistically explore the potential for a submerged macrophyte, *Hydrilla verticillata*, to influence snail densities by providing potential refugia for snails, and providing an alternative resource for the omnivorous crayfish predator, *Procambarus alleni*.

We established 70 outdoor freshwater ponds in 800-L mesocosms filled with 500 L of water at a facility approximately 20 miles southeast of Tampa, FL, USA. Tanks were set up as previously described for the mesocosm experiment, with the addition of 5 rooted shoots of *Hydrilla verticillata* added to the sediment of each tank on 5 July 2011. Immediately before application of agrochemical treatments on 11 July 2011 (Week 0), snails (21 *Bi. glabrata* (NMRI strain), and 12 *Bu. truncatus* (Egyptian strain), provided by NIAID Schistosomiasis Resource Center) and snail predators (2 juvenile crayfish (*Procambarus alleni*), 10 giant water bugs (7 *Belostoma flumineum* and 3 *Lethocerus* sp.) collected from local ponds) were added to each tank. The mesocosm experiment was approved under USF Institutional Biosafety Committee Study number 0971, with the same biosafety precautions as described above.

Tanks were randomly assigned to one of fourteen treatments (six herbicides at their respective EEC, six insecticides at their respective EEC, solvent control (0.0625 mL/L acetone), and water control) in five replicated spatial blocks. All pesticides were dissolved in acetone and applied at their respective estimated peak environmental concentrations as described above.

Data were collected at intervals as described above for the previous mesocosm experiment until the conclusion of the experiment after 12 weeks on 31 September 2011).

Because herbicides either eliminated or reduced growth of *H. verticillata* but had no apparent direct effects on invertebrate predators and insecticides eliminated invertebrate predators but had no direct effects on *H. verticillata*, we opportunistically explored the effects of *H. verticillata* and predator presence or absence on snail densities at the end of the experiment. Fixed main effects of *H. verticillata* biomass at the end of the experiment, *P. alleni* presence/absence at the end of the experiment, spatial block, and all interactions were used as predictors of the count of adult *Bi. glabrata* or *Bu. truncatus* in each tank at the end of the experiment, using a generalized linear model with a Poisson error distribution in R[1].

There were significant main effects and a significant interaction between *H. verticillata* biomass and crayfish presence on the densities of both snail species (Supplementary Fig. 4, Supplementary Table 8). For both *Bi. glabrata* and *Bu. truncatus,* crayfish presence had a strong negative effect on adult snail densities at the end of the experiment. The densities of both snail species increased with increasing *H. verticillata* biomass in the absence of crayfish, but this effect disappeared or reversed in the presence of crayfish (Supplementary Fig. 4). These results suggest that *H. verticillata* serves as an additional substrate for epiphytic algae that snails consume, and the macrophyte did not alter crayfish predation rates on snails (Supplementary Fig. 4). In fact, there was no recruitment to the adult snail population when crayfish were present regardless of the density of the macrophyte.

Both crayfish (*Procambarus* spp.) and prawns (*Macrobrachium* spp.) are omnivorous species that consume large amounts of living and decaying plant and algal matter in addition to a wide variety of animal prey[2–9]. Therefore, the presence of alternative food resources could

impact the strength of the top-down effects on snail densities[9]. We attempted to avoid forcing crayfish to consume only *Schistosoma*-harboring snail hosts in the first mesocosm experiment by adding a third, non-host snail species. In this separate mesocosm experiment, *Hydrilla verticillata* served as an alternative food resource for crayfish, yet it did not limit the effects of predation on snail densities (Supplementary Fig. 4), suggesting that at least this macrophyte does not reduce the strength of top-down effects of crayfish on these snail populations and that our results are robust to the presence of alternative food resources for omnivorous snail predators. Additionally, our mathematical model (see *Modelling Experiments* in the Methods of the main text) implicitly accounts for prey-switching behavior of generalist snail predators by the use of a Holling type III functional response, which specifies that the per-capita, per-predator predation mortality of the snails is not constant but rather increases with snail density to represent how a predator's preference switches away from snails when snail abundance is low. Finally, field research in Senegal involving the reintroduction of *M. vollenhovenii* to natural waterbodies, which presumably included a much wider variety of available food resources for prawns, found very similar effects on the densities of *Bulinus truncatus* and *Bu. globosus*, reinforcing our conclusion that the top-down effects of snail predators are very strong in natural settings when predators are present.

The potential effects of refugia on final snail densities (and infected snail densities) are less clear from our results. Although submerged macrophytes might provide snails with a potential refuge from predators[10], there was no evidence from the mesocosm experiment that *H. verticillata* increased snail recruitment when crayfish were present. However, the effects of refugia on snail population dynamics in natural environments are complex because refuge-seeking behaviors, and growth and reproduction rates of snails can be modified by the types and

densities of predators, snail densities, resource availability, and snail infection status[11–15]. While a greater availability of refugia in the mesocosm experiments would likely have resulted in higher overall snail densities, the net effects of refugia on infected snail densities are more difficult to predict because prawns (and perhaps crayfish) show a preference for consuming schistosome-infected *Bi. glabrata* and *Bu. truncatus* in predation trials, and infected *Bi. glabrata* and *Bu. truncatus* exhibit less frequent and slower movement and lower refuge use (above water or under substrate) than uninfected snails in response to predation cues in laboratory trials[15]. The effects and interactions of refuge availability, resource availability (especially as modified by agrochemical contamination), and multiple types of predators on schistosome-infected snail densities clearly deserves further examination. However, while these factors are might affect the relative strengths of bottom-up vs. top-down regulation of infected snail densities, multiple field observations and manipulations in natural settings[2,4,5,8,16–18] are consistent with our experimental and modelling results that highlight the importance of predators in regulating snail densities.

While food web theory provides an excellent basis for predicting the combined effects of agrochemicals on biodiversity and ecosystem functions in mesocosm experiments[19], field trials are necessary to examine community-level responses in open systems that can vary in snail species composition and in which the recruitment and recovery of predator populations can be estimated. The effects of predators and agrochemicals on aquatic biodiversity *in situ*, and how such changes in local biodiversity might then influence transmission dynamics, also deserves greater attention. In general, declines in local biodiversity caused by agrochemical contamination might be expected to increase infection rates through the dilution effect[20], where decreases in the predators of snails (as we show here) and/or predators of free-swimming schistosome miracidia/cercariae can indirectly increase exposure by increasing the densities of host snail

species and/or miracidia[18]. However, the opposite might be true if non-schistosome host snail species that could potentially act as decoys for schistosome miracidia[21,22] experience relatively higher increases in abundance than do schistosome host snails. We included two non-host snail species for each species of schistosome in our mesocosm experiment to account for the decoy effect, but the degree to which the richness and identity of non-host snail species in the field might influence the magnitude of the effect sizes observed in our study remains to be seen.

Code Availability

Scripts containing the code used for data analysis in R are available from the corresponding author upon request.

Data Availability

The experimental data that support the findings of the supplementary experiment are publicly available in figshare with the identifier doi:10.6084/m9.figshare.5797389

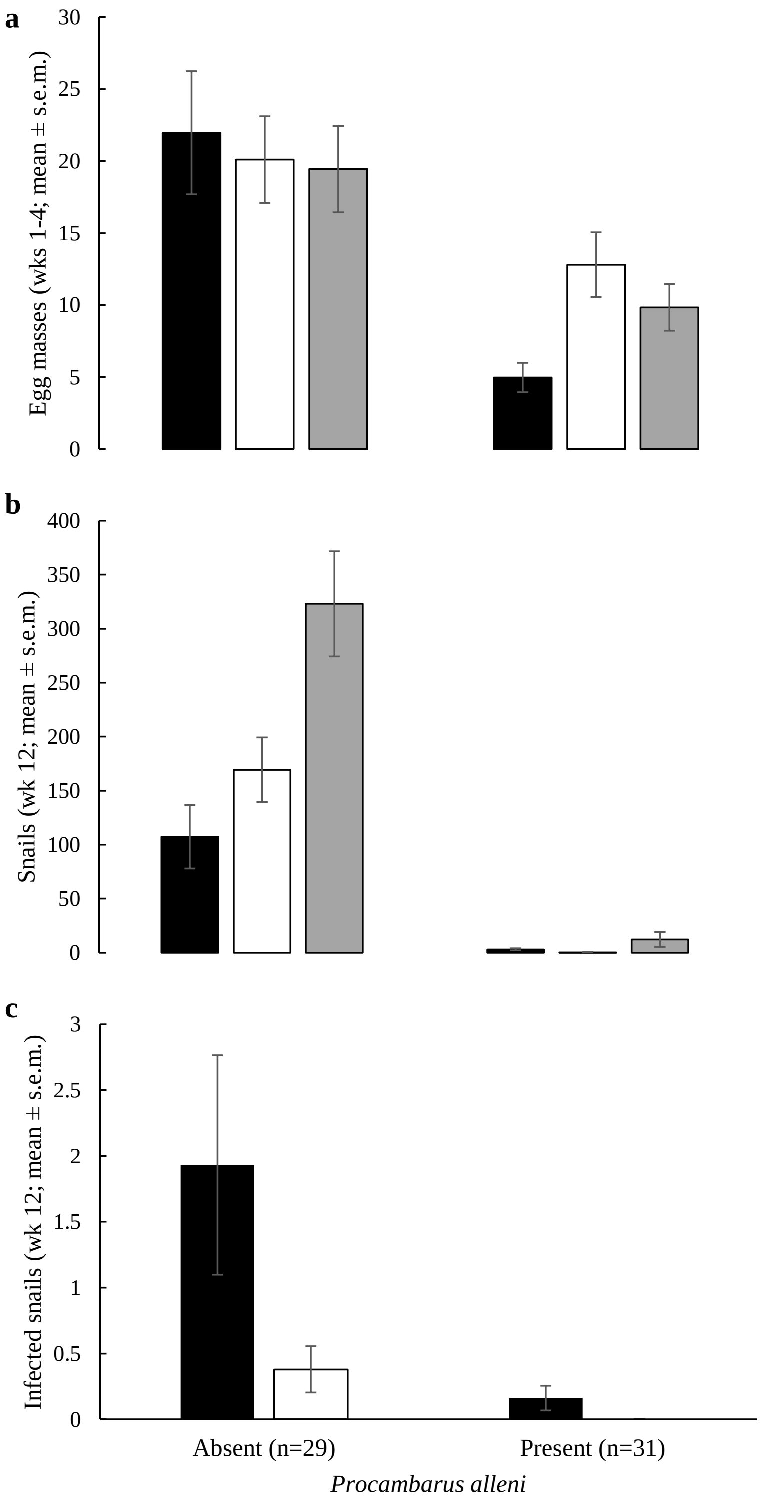

Supplementary Figure 1. Effects of predator (*Procambarus alleni*) presence or absence (measured at the end of the experiment) on snail reproductive effort (**a**), final snail densities (**b**), and infected snail densities (**c**) for each species of snail. Black bars represent *Bi. glabrata*, white bars represent *Bu. truncatus*, and gray bars indicate *H. cubensis*, which cannot be infected and thus are not included in **c**.

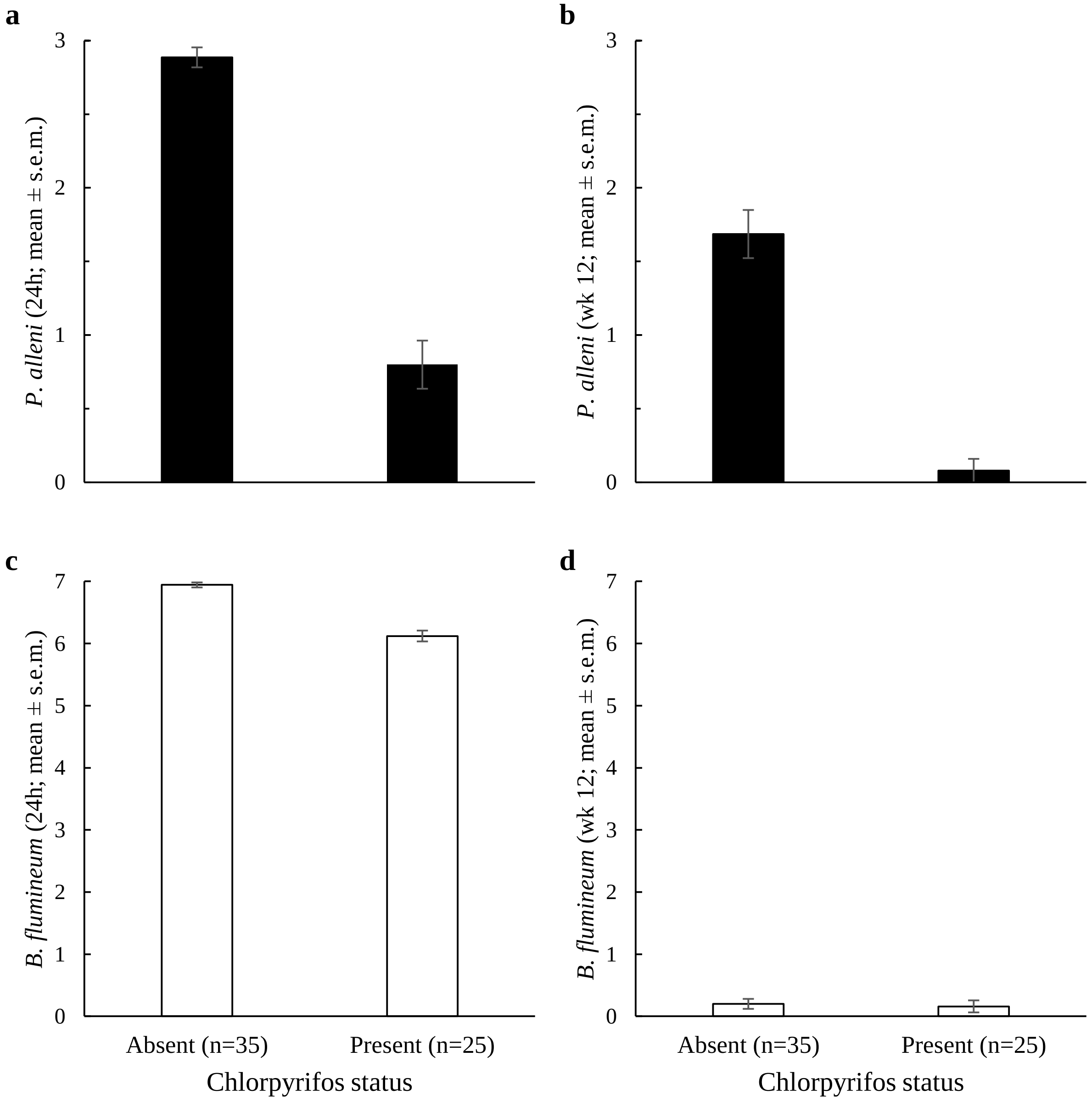

Supplementary Figure 2. Effects of chlorpyrifos treatment on survival of *Procambarus alleni* (black bars; **a,b**) and *Belostoma flumineum* (white bars; **c,d**) 24 h after exposure to chlorpyrifos (**a,c**) and at the conclusion of the experiment (**b,d**).

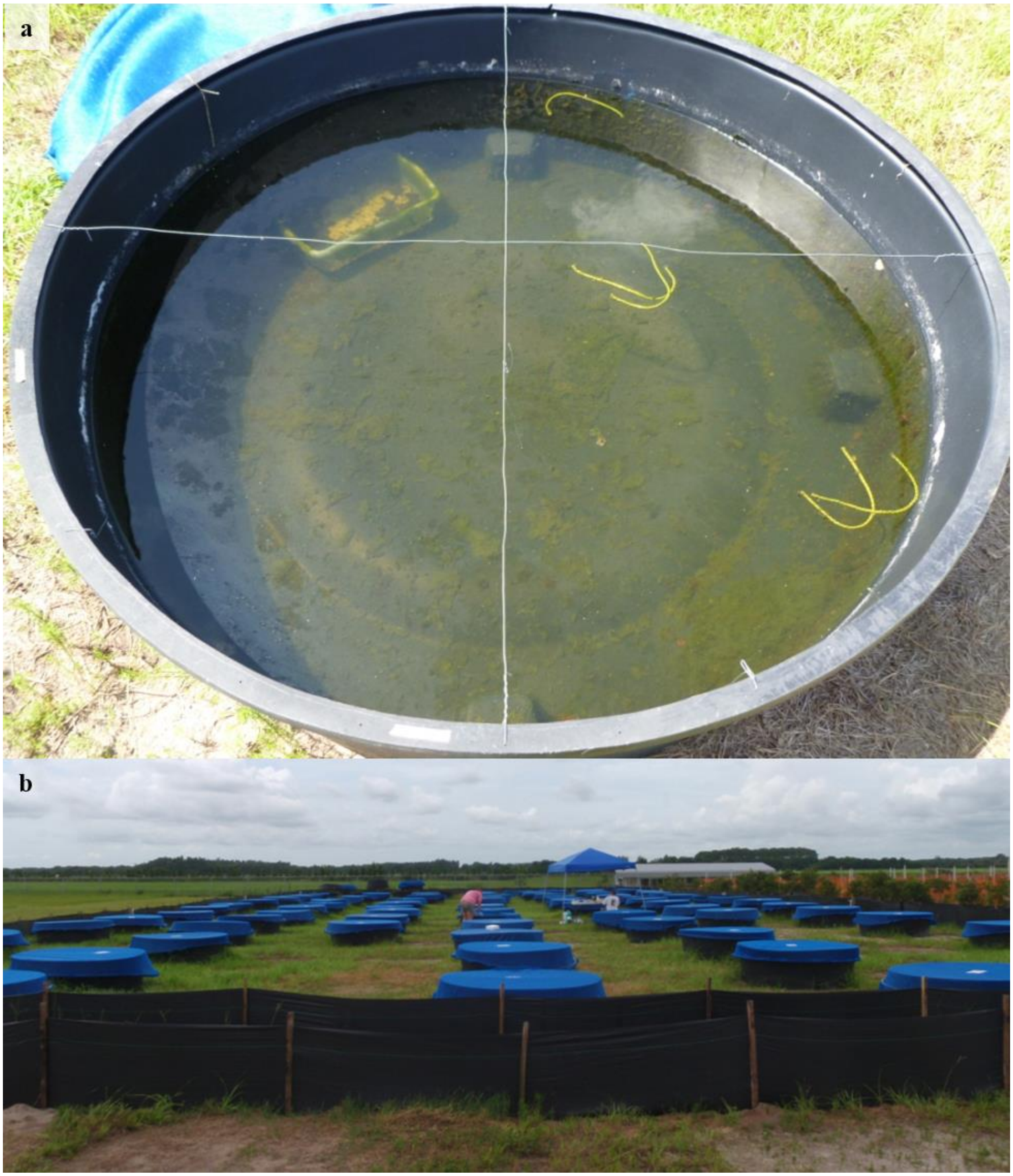

Supplementary Figure 3. Photographs of a similar mesocosm setup, showing an example mesocosm just prior to the addition of snails, snail predators, and agrochemical treatments (**a**) and the outdoor facility with a double layer of silt fence surrounding the tanks (**b**). The actual mesocosms for this study consisted of a slightly smaller tank nested within a larger tank to which water and pool shock (71.8% trichloro-s-triazinetrione) were added to kill any potential snails escaping from the inner tank. In addition, molluscicide (1.0% iron phosphate) was applied to the area between the silt fences to act as an additional barrier to snails. Photographs **a** and **b** taken by Neal Halstead.

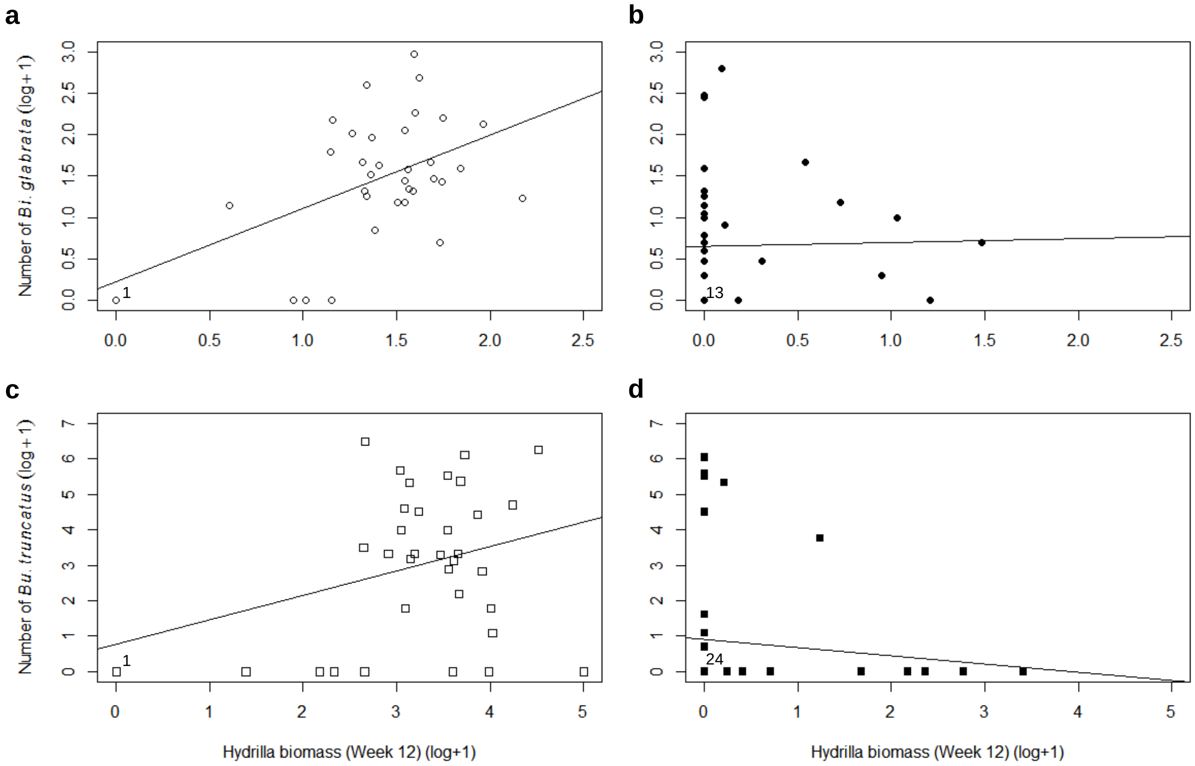

Supplementary Figure 4. Final densities of *Biomphalaria glabrata* (circles; **a,b**) and *Bulinus truncatus* (squares; **c,d**) in response to the biomass of *Hydrilla verticillata* and predator absence (open symbols; **a,c**) or presence (solid symbols; **b,d**) at the end of a separate mesocosm experiment that included submerged macrophytes as a refugia and food source for snails and an alternative food source for omnivorous crayfish *Procambarus alleni*. For both species of snails, density increased with increasing biomass of *Hydrilla* when crayfish were absent from mesocosms, but not in the presence of predators. Thus, the density of this macrophyte species did not significantly affect the interaction strength between this crayfish predator and either *Bi. glabrata* or *Bu. truncatus*. Numbers next to 0,0 points indicate the number of replicates with both snails and *Hydrilla* absent.

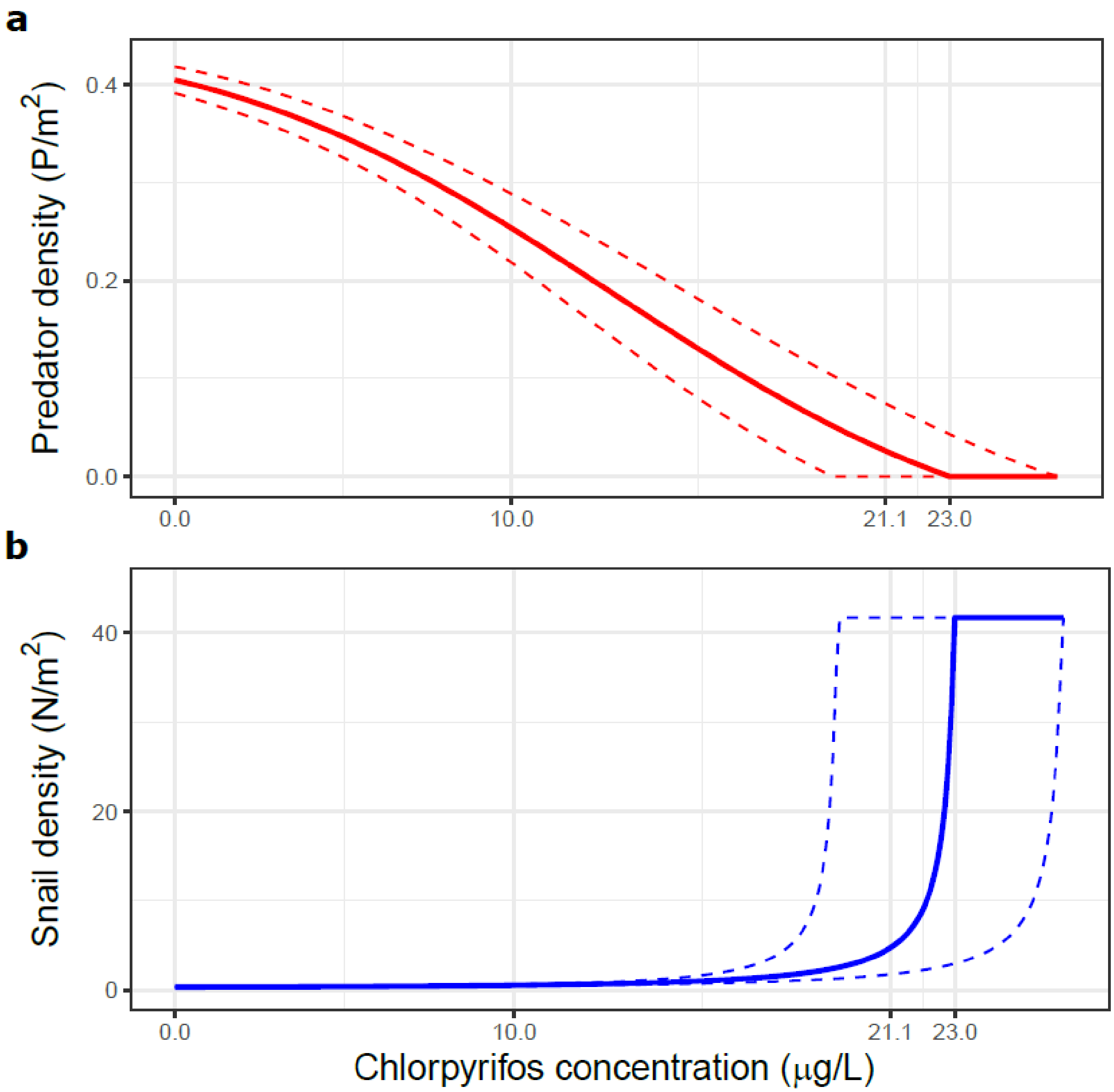

Supplementary Figure 5. Equilibrium predator (**a**) and snail (**b**) densities at chlorpyrifos concentrations <25 μg/L. Interactions between these populations determine R_0 at concentrations <23.0 μg/L whereas predator elimination—and endemic transmission equivalent to that in a predator-free setting—is expected to occur at concentrations >23.0 μg/L. At chlorpyrifos concentrations >21.6 μg/L, predator mortality is high enough to allow sufficient snail reproduction to occur, causing R_0 >1.

Supplementary Table 1. Results of the structural equation model conducted in the package lavaan in R revealing the relationships among agrochemical mixtures, predator density, algal productivity, and snail density. Densities of all three snail species, including measures of reproductive effort (eggs and hatchlings) contributed significantly to the latent variable “snail density” and generally covaried positively, emphasizing the similarity of treatment effects across all snail species.

Latent variables	Estimate	Std.err	Standardized estimate	Z-value	P (>|z|)
Predator mortality					
P. alleni (24h)	1.000		0.874		
P. alleni (end)	0.580	0.098	0.641	5.934	**<0.001**
B. flumineum (24h)	0.532	0.066	0.779	8.018	**<0.001**
B. flumineum (end)	0.004	0.016	0.032	0.247	0.805
Phytoplankton					
F_0 (week 1)	1.000		0.361		
F_0 (week 2)	1.583	0.609	0.791	2.601	**0.009**
F_0 (week 4)	1.836	0.859	0.436	2.137	**0.033**
F_0 (week 8)	1.634	0.954	0.296	1.713	*0.087*
QY (week 1)	2.140	0.977	0.243	2.191	**0.028**
QY (week 2)	6.357	2.443	0.793	2.602	**0.009**
QY (week 4)	3.942	1.832	0.443	2.152	**0.031**
QY (week 8)	4.602	2.266	0.394	2.031	**0.042**
Periphyton F_0					
F_0 (week 1)	1.000		0.560		
F_0 (week 2)	0.966	0.296	0.536	3.262	**0.001**
F_0 (week 4)	0.900	0.345	0.403	2.612	**0.009**
Periphyton QY					
QY (week 1)	1.000		0.300		
QY (week 2)	3.555	2.064	0.431	1.722	*0.085*
QY (week 4)	2.235	1.233	0.506	1.813	*0.070*
Snail density					
Bi. glabrata eggs	1.000		0.722		
Bi. glabrata hatch.	6.243	1.373	0.583	4.548	**<0.001**
Bi. glabrata adults	1.756	0.287	0.776	6.120	**<0.001**
Bu. truncatus eggs	0.478	0.131	0.391	3.476	**0.001**
Bu. truncatus hatch.	1.536	0.199	0.963	7.703	**<0.001**
Bu. truncatus adults	2.732	0.353	0.966	7.732	**<0.001**
H. cubensis eggs	0.574	0.118	0.487	4.884	**<0.001**
H. cubensis hatch.	1.431	0.215	0.842	6.671	**<0.001**
H. cubensis adults	2.808	0.384	0.918	7.309	**<0.001**

Composite variables	Estimate	Std.err	Standardized estimate	Z-value	P (>|z|)
Algal production					
Phytoplankton	1.000		1.149		

Periphyton F0	-0.044	0.138	-0.111	-0.321	0.749
Periphyton QY	1.705	0.635	0.999	2.685	**0.007**
Covariances	Estimate	Std.err	Standardized estimate	Z-value	P (>|z|)
Predator mortality					
B. flumineum (24h) ~ *B. flumineum* (end)	0.005	0.002	0.335	2.507	**0.012**
Phytoplankton					
F_0(wk 1) ~ QY(wk 1)	0.328	0.085	0.589	3.841	**<0.001**
F_0(wk 4) ~ QY(wk 4)	0.544	0.128	0.712	4.263	**<0.001**
F_0(wk 8) ~ QY(wk 8)	0.819	0.219	0.573	3.733	**<0.001**
Snail density					
*Bu. trunc*atus eggs ~ *H. cubensis* eggs	0.006	0.001	0.637	4.145	**0.000**
Bi. glabrata eggs ~ *Bu. truncatus* eggs	0.004	0.001	0.478	3.309	**0.001**
Bi. glabrata eggs ~ *H. cubensis* eggs	0.003	0.001	0.343	2.490	**0.013**
Bu. truncatus hatch ~ *H. cubensis* end	-0.002	0.001	-0.558	-3.348	**0.001**
Bi. glabrata hatch ~ *H. cubensis* hatch	0.025	0.009	0.408	2.867	**0.004**
Periphyton F_0 ~ Periphyton QY	-0.001	0.000	-0.396	-2.853	**0.004**
Regressions	Estimate	Std.err	Standardized estimate	Z-value	P(>|z|)
Predator mortality~					
Chlorpyrifos	1.050	0.008	0.998	133.140	**<0.001**
Phytoplankton~					
Atrazine	-0.059	0.022	-0.201	-2.735	**0.006**
Fertilizer	0.234	0.022	0.793	10.806	**<0.001**
Phytoplankton F_0~					
Atrazine	-0.274	0.027	-0.424	-10.303	**<0.001**
Fertilizer	0.330	0.025	0.510	13.104	**<0.001**
At*Fe	0.571	0.037	0.692	15.597	**<0.001**
Periphyton QY~					
Atrazine	0.123	0.006	0.816	19.751	**<0.001**
Fertilizer	-0.069	0.006	-0.458	-11.082	**<0.001**
Snail density~					
Predator mortality	0.164	0.021	0.977	7.838	**<0.001**
Algal production	0.081	0.029	0.118	2.780	**<0.001**

Supplementary Table 2. Results of a zero-inflated Poisson model on density of infected *Biomphalaria glabrata* at the end of the experiment. The zero-inflated portion of the model included only the intercept and crayfish survival as no other predictor variables were significant and model selection indicated the simpler model was a better fit (ΔAICc >2). Analysis was performed only for infected *Bi. glabrata* as too few infected *Bu. truncatus* were present at the end of the experiment. At = atrazine; Ch = chlorpyrifos; Fe = fertilizer.

Count model coefficients (Poisson with log link)

Term	Coefficient	Std. Error	z-value	P
intercept	-0.23	1.14	-0.20	0.842
Bi. glabrata density	0.007	0.002	4.50	**<0.001**
P. alleni survival	3.03	3.84	0.79	0.429
Block2	2.17	1.12	1.93	0.054
Block3	-3.58	7.61	-0.47	0.638
Block4	2.47	1.12	2.20	**0.028**
Block5	0.36	1.43	0.25	0.802
At	-2.38	1.70	-1.40	0.162
Ch	-1.74	1.51	-1.16	0.247
Fe	-17.09	145.05	-0.12	0.906
At:Ch	2.43	1.79	1.36	0.175
At:Fe	16.89	144.90	0.12	0.907
Ch:Fe	16.16	145.05	0.11	0.911
At:Ch:Fe	-16.38	144.90	-0.11	0.910

Zero-inflation model coefficients (binomial with logit link)

Term	Coefficient	Std. Error	z-value	P
Intercept	-1.66	0.83	-2.00	**0.046**
P. alleni survival	1.78	0.58	3.09	**0.002**

Supplementary Table 3. Results of generalized linear mixed model on infection prevalence of *Schistosoma mansoni* in *Biomphalaria glabrata*. Number of infected *Bi. glabrata* versus number of uninfected *Bi. glabrata* in each replicate was modeled using a beta binomial distribution and fixed effects of all predictor variables. At = atrazine; Ch = chlorpyrifos; Fe = fertilizer.

Term	Coefficient	Std. Error	z-value	P
intercept	-3.85	1.27	-3.03	**0.002**
At	0.36	1.29	0.28	0.780
Ch	-0.32	1.14	-0.28	0.777
Fe	-12.48	1712.60	-0.01	0.994
At:Ch	-0.28	1.47	-0.19	0.847
At:Fe	12.75	1712.60	0.01	0.994
Ch:Fe	11.48	1712.60	0.01	0.995
At:Ch:Fe	-11.94	1712.60	-0.01	0.994
Block 2	0.69	0.86	0.80	0.424
Block 3	0.49	1.03	0.47	0.635
Block 4	0.19	0.91	0.21	0.831
Block 5	0.20	0.90	0.23	0.822

Supplementary Table 4. Results of generalized linear mixed model on *S. mansoni* cercaria shedding rates from infected *Bi. glabrata*. The number of cercariae shed per hour was modeled using a negative binomial distribution. At = atrazine; Ch = chlorpyrifos; Fe = fertilizer.

Main effects of agrochemicals				
Term	Coefficient	Std. Error	z-value	P
Intercept	-0.498	1.549	-0.32	0.750
Days post-exposure	0.138	0.035	4.01	**<0.001**
At	-0.175	0.275	-0.64	0.520
Ch	-0.014	0.315	-0.04	0.970
Fe	-0.106	0.274	-0.39	0.700

Interaction between atrazine and fertilizer				
Term	Coefficient	Std. Error	z-value	P
Intercept	-1.262	1.959	-0.64	0.519
Days post-exposure	0.161	0.047	3.45	**<0.001**
At	-0.341	0.370	-0.92	0.356
Fe	-0.321	0.394	-0.81	0.415
At:Fe	0.409	0.564	0.73	0.468

Supplementary Table 5. Mean percent of *Schistosoma mansoni* cercariae that were dead (s.e.m. in parentheses) after 2, 4, 8, 12, or 24 h of exposure to solvent control or the estimated environmental concentration of atrazine, chlorpyrifos, or fertilizer (see Methods for actual concentrations; n = 6). Significant treatment effects are in bold. There were no significant effects of treatment on cercarial survival when including time since agrochemical exposure as a predictor variable. When analyzing data from each time point independently, there were no significant effects of treatment at or before 12 h. Given that *S. mansoni* are only infective for approximately 12 h[23], survival differences after this time period are less ecologically relevant than before it.

Treatment	2 h	4 h	8 h	12 h	24 h	Mean
Atrazine	2.78 (2.78)	8.33 (8.33)	4.83 (3.88)	3.27 (2.58)	74.07 (10.21)	19.13 (5.96)
Chlorpyrifos	5.56 (5.56)	1.39 (1.39)	4.78 (3.36)	1.52 (1.52)	**86.30 (5.34)**	19.91 (6.38)
Fertilizer	0.00 (0.00)	1.19 (1.19)	6.22 (4.06)	9.52 (7.06)	**85.57 (7.83)**	20.99 (6.62)
Solvent	2.38 (2.38)	1.85 (1.85)	1.39 (1.39)	12.00 (4.66)	55.56 (15.91)	14.63 (4.98)

Supplementary Table 6. Results of generalized linear mixed model on *S. mansoni* and *S. haematobium* egg viability. The number of hatched eggs was modeled using a beta binomial distribution. At = atrazine; Ch = chlorpyrifos; Fe = fertilizer.

	Schistosoma mansoni			
Term	Coefficient	Std. Error	z-value	P
Intercept	-2.301	0.230	-10.02	<0.001
At	-0.109	0.272	-0.40	0.689
Ch	-0.294	0.289	-1.02	0.310
Fe	0.009	0.262	0.03	0.973
At:Ch	0.506	0.418	1.21	0.226
At:Fe	0.358	0.404	0.88	0.376
Ch:Fe	0.037	0.453	0.08	0.936
At:Ch:Fe	-1.333	0.701	-1.90	0.057

	Schistosoma haematobium			
Term	Coefficient	Std. Error	z-value	P
Intercept	-3.798	0.884	-4.92	<0.001
At	0.066	0.431	0.15	0.880
Ch	-0.175	0.455	-0.38	0.700
Fe	-0.200	0.467	-0.43	0.670
At:Ch	0.761	0.643	1.18	0.240
At:Fe	-0.701	0.790	-0.89	0.370
Ch:Fe	1.159	0.920	-1.26	0.210
At:Ch:Fe	1.144	1.255	0.91	0.360

Supplementary Table 7. Parameters used for calculation of peak estimated environmental concentrations (EECs).

GENEEC Parameter	Atrazine	Chlorpyrifos
Trade name	Aatrex	Dursban 50W
Crop	Corn	Turfgrass
Rate (pounds of active ingredients/acre taken from specimen label)	2	8
Number of applications	1	1
Times between applications	-	-
koc (use lowest)	100[b]	6070[b]
Soil half-life (days)	300[c]	30.5[c]
Wetted application?	No	No
Application method	Ground spray[a]	Ground spray[a]
Nozzle height (in.)	20-50: EFED[a]	20-50: EFED[a]
Spray Quality	fine: EFED[a]	fine: EFED[a]
No spray zone (feet)	0	0
Depth of incorporation (0-6 inches)	0	0
Solubility (mg/L)	33	2[b]
Aquatic half-life (days) - use longest	742[d]	-
Hydrolysis half-life (days) - use longest	-	78[d]
Photolysis half-life (days) - usually the longest number	335[d]	28[d]
Peak EEC (µg/L)	102	64
Actual concentration (µg/L)	99	NA[e]

[a] – Program default value
[b] – http://extoxnet.orst.edu/
[c] – USDA
[d] – Spectrum Laboratories
[e] – Absorbances of diluted samples for chlorpyrifos were outside range of standard solutions in ELISA assay, so nominal concentrations were used for analyses

Supplementary Table 8. Results of generalized linear model on *Bi. glabrata* and *Bu. truncatus* densities in response to *Hydrilla verticillata* biomass and the presence or absence of crayfish predators. The number of snails in each mesocosm at the end of the experiment was modeled using a Poisson distribution with interaction terms between the biomass of *Hydrilla* in each tank at the end of the experiment, the presence or absence of predators, their interaction, and spatial block.

	Biomphalaria glabrata			
Term	Coefficient	Std. Error	z-value	P
Intercept	4.098	0.045	90.90	<0.001
Hydrilla biomass	0.007	<0.001	8.60	<0.001
Crayfish presence	-0.966	0.045	-21.59	<0.001
*Hydrilla**crayfish	-0.083	0.015	-5.41	<0.001
Block2	0.297	0.053	5.57	<0.001
Block3	0.124	0.054	2.29	0.022
Block4	1.244	0.047	26.63	<0.001
Block5	-1.532	0.086	-17.71	<0.001

	Bulinus truncatus			
Term	Coefficient	Std. Error	z-value	P
Intercept	4.614	0.034	134.80	<0.001
Hydrilla biomass	0.014	<0.001	19.80	<0.001
Crayfish presence	-0.642	0.043	-15.08	<0.001
*Hydrilla**crayfish	-0.442	0.051	-8.68	<0.001
Block2	0.102	0.040	2.54	0.011
Block3	-0.805	0.048	-16.83	<0.001
Block4	-0.416	0.045	-9.26	<0.001
Block5	-2.778	0.095	-29.16	<0.001

Supplementary Table 9. Model parameter symbology, definitions, values and sources used as reference literature for parameter values.

Symbol	Definition	Value	Source
f_N	Per-capita daily fertility rate of snails including survival to detectability	0.10	[24]
φ_N	Density-dependent snail population parameter, roughly the inverse of snail carrying capacity	10^3 (~50/m^2)	[24]
$\varphi_{N,q}$	Scalar of the density dependent snail population parameter caused by fertilizer and/or atrazine stimulation of algal resources	See Supplementary Table 10	This study
μ_N	Natural per-capita daily mortality rate of snails	0.017	[24]
β	Infection probability from man to snail; interpreted as the per-capita daily probability of snail infection given the number of mated female worms (*M*)	1.63x10^{-5}	Fit to epi data
σ	Per-capita daily conversion rate of exposed to infected snails	0.025	[25]
n	Exponent of prey density in Holling III functional response	2	[26]
μ_I	Additional per-capita daily mortality of infected snails	0.083	[25]
λ_{lo}	Infection probability from snail to man in low transmission season; interpreted as the per-capita daily probability an adult worm establishes within a human host given the number of shedding snails	3.67x10^{-6}	Fit to epi data
λ_{hi}	Infection probability from snail to man in high transmission season	2.45x10^{-4}	Fit to epi data
μ_H	Per-capita daily mortality rate of adult worms caused by human mortality (assuming lifespan of 60 years)	4.57x10^{-5}	[5]
H	Total human population interacting with water contact site	300	[5]
k	Clumping parameter of the negative binomial distribution of worms within the human population	0.08	Estimated from epi data
μ_W	Natural per-capita daily mortality rate of adult worms (assuming lifespan of 3.3 years)	8.3x10^{-4}	[27]
f_P	Per-capita daily fertility rate of predator population including survival to effective snail predation	0.117	[28]
φ_P	Predator carrying capacity	120 (~0.6/m^2)	This study
μ_P	Natural per-capita daily predator mortality rate	See Supplementary Table 10	This study
$\mu_{P,q}$	Additional per-capita daily mortality rate caused by insecticide at concentration, *q*	See Supplementary Table 10	[29]
α	Per capita attack rate of predators on snails at low densities	0.003	[6]
T_h	Predation saturation parameter; approximately the inverse of the daily maximum snails consumed per *Procambarus clarkii* predator	0.067	[30]

Supplementary Table 10. Model parameter distributions included in the Monte Carlo simulation.

Symbol	Distribution	Agrochemical Treatment	Distribution parameters
$\varphi_{N,q}$	Normal	Fertilizer	Mean = 1.16<br>St. dev = 0.52
		Atrazine	Mean = 1.63<br>St. dev = 0.46
		Atrazine & Fertilizer	Mean = 1.50<br>St. dev = 0.28
μ_P	Beta	Chlorpyrifos absent	α = 2.81<br>β = 70.22
$\mu_{P,q}$		Chlorpyrifos present	α = 50.39<br>β = 18.28
β	Weighted by likelihood	NA	95% CI = $1.63 \times 10^{-6} – 3.11 \times 10^{-5}$
λ_{lo}		NA	95% CI = $3.67 \times 10^{-7} – 6.97 \times 10^{-6}$
λ_{hi}		NA	95% CI = $9.51 \times 10^{-4} – 4.66 \times 10^{-4}$

Supplementary References

1. R Core Team. R: A language and environment for statistical computing. (2015).

2. Mkoji, G. M. *et al.* Impact of the crayfish *Procambarus clarkii* on *Schistosoma haematobium* transmission in Kenya. *Am. J. Trop. Med. Hyg.* **61,** 751–759 (1999).

3. Hofkin, B. V, Koech, D. K., Oumaj, J. & Loker, E. S. The North American crayfish *Procambarus clarkii* and the biological control of schistosome-transmitting snails in Kenya: Laboratory and field investigations. *Biol. Control* **1,** 183–187 (1991).

4. Savaya Alkalay, A. *et al.* The prawn *Macrobrachium vollenhovenii* in the Senegal River Basin: towards sustainable restocking of all-male populations for biological control of schistosomiasis. *PLoS Negl. Trop. Dis.* **8,** e3060 (2014).

5. Sokolow, S. H. *et al.* Reduced transmission of human schistosomiasis after restoration of a native river prawn that preys on the snail intermediate host. *Proc. Natl. Acad. Sci. U. S. A.* **112,** 9650–9655 (2015).

6. Sokolow, S. H., Lafferty, K. D. & Kuris, A. M. Regulation of laboratory populations of snails (*Biomphalaria* and *Bulinus* spp.) by river prawns, *Macrobrachium* spp. (Decapoda, Palaemonidae): implications for control of schistosomiasis. *Acta Trop.* **132,** 64–74 (2014).

7. Dorn, N. J. & Wojdak, J. M. The role of omnivorous crayfish in littoral communities. *Oecologia* **140,** 150–9 (2004).

8. Khalil, M. T. & Sleem, S. H. Can the freshwater crayfish eradicate schistosomiasis in Egypt and Africa ? *J. Am. Sci.* **7,** 457–462 (2011).

9. Marçal, A. *et al.* Predator–prey interactions of *Procambarus clarkii* with aquatic macroinvertebrates in single and multiple prey systems. *Acta Oecologica* **28,** 337–343 (2005).

10. Bronmark, C. Interactions between epiphytes, macrophytes and freshwater snails: a review. *J. Molluscan Stud.* **55,** 299–311 (1989).

11. Turner, A. M., Bernot, R. J. & Boes, C. M. Chemical cues modify species interactions: the ecological consequences of predator avoidance by freshwater snails. *Oikos* **88,** 148–158 (2000).

12. Turner, A. M., Turner, S. E. & Lappi, H. M. Learning, memory and predator avoidance by freshwater snails: effects of experience on predator recognition and defensive strategy. *Anim. Behav.* **72,** 1443–1450 (2006).

13. Turner, A. M. Non-lethal effects of predators on prey growth rates depend on prey density and nutrient additions. *Oikos* **104,** 561–569 (2004).

14. Hoverman, J. T., Auld, J. R. & Relyea, R. A. Putting prey back together again: integrating predator-induced behavior, morphology, and life history. *Oecologia* **144,** 481–91 (2005).

15. Swartz, S. J., De Leo, G. A., Wood, C. L. & Sokolow, S. H. Infection with schistosome parasites in snails leads to increased predation by prawns: implications for human schistosomiasis control. *J. Exp. Biol.* **218,** 3962–3967 (2015).

16. Evers, B. N., Madsen, H., Alle, J. & Road, B. The schistosome intermediate host, *Bulinus nyassanus*, is a 'preferred' food for the cichlid fish, *Trematocranus placodon*, at Cape Maclear , Lake Malawi. *Ann. Trop. Med. Parasitol.* **100,** 75–85 (2006).

17. Madsen, H. & Stauffer, J. R. Density of Trematocranus placodon (Pisces: Cichlidae): A predictor of density of the schistosome intermediate host, Bulinus nyassanus (Gastropoda: Planorbidae), in Lake Malawi. *Ecohealth* **8,** 177–189 (2011).

18. Rohr, J. R. *et al.* Predator diversity, intraguild predation, and indirect effects drive parasite transmission. *Proc. Natl. Acad. Sci. U. S. A.* **112,** 3008–3013 (2015).

19. Halstead, N. T. *et al.* Community ecology theory predicts the effects of agrochemical mixtures on aquatic biodiversity and ecosystem properties. *Ecol. Lett.* **17,** 932–941 (2014).

20. Civitello, D. J. *et al.* Biodiversity inhibits parasites: Broad evidence for the dilution effect. *Proc. Natl. Acad. Sci. U. S. A.* **112,** 8667–8671 (2015).

21. Johnson, P. T. J., Lund, P. J., Hartson, R. B. & Yoshino, T. P. Community diversity reduces *Schistosoma mansoni* transmission, host pathology and human infection risk. *Proc. R. Soc. B-Biological Sci.* **276,** 1657–1663 (2009).

22. Johnson, P. T. J. & Thieltges, D. W. Diversity, decoys and the dilution effect: how ecological communities affect disease risk. *J. Exp. Biol.* **213,** 961–70 (2010).

23. Olivier, L. J. Infectivity of *Schistosoma mansoni* cercariae. *Am. J. Trop. Med. Hyg.* **15,** 882–885 (1966).

24. Woolhouse, M. E. J. & Chandiwana, S. K. Population biology of the freshwater snail *Bulinus globosus* in the Zimbabwe highveld. *J. Appl. Ecol.* **27,** 41–59 (1990).

25. Anderson, R. M. & May, R. M. *Infectious Diseases of Humans*. (Oxford University Press, 1991).

26. Real, L. A. The kinetics of functional response. *Am. Nat.* **111,** 289–300 (1977).

27. Goddard, M. J. & Jordan, P. On the longevity of *Schistosoma mansoni* in man on St. Lucia, West Indies. *Trans. R. Soc. Trop. Med. Hyg.* **74,** 185–191 (1980).

28. Cervantes-Santiago, E., Hernández-Vergara, M. P., Pérez-Rostro, C. I. & Olvera-Novoa, M. A. Reproductive performance of the crayfish *Procambarus* (*Austrocambarus*) *acanthophorus* Villalobos 1948 under controlled conditions. *Aquaculture* **308,** 66–70 (2010).

29. Halstead, N. T., Civitello, D. J. & Rohr, J. R. Comparative toxicities of organophosphate and pyrethroid insecticides to aquatic macroarthropods. *Chemosphere* **135,** 265–271 (2015).

30. Hofkin, B. V, Mkoji, G. M., Koech, D. K. & Loker, E. S. Control of schistosome-transmitting snails in Kenya by the North American crayfish *Procambarus clarkii*. *Am. J. Trop. Med. Hyg.* **45,** 339–44 (1991).
